# Heart transplantation in a patient with infective endocarditis bridged with Impella 5.5: a case report

**DOI:** 10.1093/ehjcr/ytae062

**Published:** 2024-03-08

**Authors:** Nadeem A Khan, Bassam Shukrullah, Peter M Eckman, Katarzyna M Hryniewicz

**Affiliations:** Section of Advanced Heart Failure and Transplant, Minneapolis Heart Institute, 800 E 28th St, Minneapolis, MN 55407, USA; School of Medicine, Southern Illinois University, Springfield, IL, USA; Section of Cardiothoracic Surgery, University of Alabama at Birmingham, Birmingham, AL, USA; Section of Advanced Heart Failure and Transplant, Minneapolis Heart Institute, 800 E 28th St, Minneapolis, MN 55407, USA; Section of Advanced Heart Failure and Transplant, Minneapolis Heart Institute, 800 E 28th St, Minneapolis, MN 55407, USA

**Keywords:** Case report, Orthotopic heart transplant, Infective endocarditis, Impella, Dilated cardiomyopathy, Aortic valve replacement

## Abstract

**Background:**

Infective endocarditis (IE) is a feared complication after surgical valve replacement accounting for 10% to 30% of all cases of IE. Our case is unique as we present a decompensated heart failure patient with IE who urgently needed mechanical circulatory support (MCS) to stabilize while IE was treated. We used Impella to bridge him to sterile state before heart transplant was done. This case highlights the importance of different strategies for bridge to heart transplant in decompensated heart failure patients with endocarditis.

**Case summary:**

We describe a case of 62-year-old male who initially presented with severe shortness of breath with minimal exertion, weight gain, and lower extremity oedema diagnosed with acute on chronic systolic heart failure (HF) exacerbation (ACC stage D, NYHA class IV). Initial blood cultures and extensive work-up for IE were negative. He continued to decompensate haemodynamically despite inotropic support and the decision was to proceed with durable left ventricular assist device (LVAD) as bridge to orthotopic heart transplantation (OHT). Immediately prior to LVAD implantation, patient’s blood cultures became positive for *Cutibacterium acnes*. Echocardiogram revealed IE on bioprosthetic aortic valve. Patient therefore underwent urgent aortic valve replacement (AVR) and was stabilized with Impella 5.5.

**Discussion:**

We highlight a case where MCS with Impella was used as a bridge to transplant in a decompensated HF patient who was septic. Patient was listed for OHT but was found to be septic due to IE and had to undergo AVR to achieve infection source control prior to undergoing heart transplant. Impella was used effectively to stabilize ACC stage D/NYHA class IV patient while he recovered from AVR and endocarditis before his blood cultures cleared up and he was listed for OHT. He successfully underwent OHT after 3 weeks.

**Learning points**

- Using mechanical circulatory support devices as a bridge while infective endocarditis or other medical comorbidities can be treated/fixed in a short amount of time, before proceeding with heart transplant.
- Using Impella assist device as an option for bridging in patients with severe heart failure/cardiogenic shock who are not candidates for left ventricular assist device/heart transplant due to active infection.

## Introduction

Infective endocarditis (IE) causes significant complications after valve replacement, ranging from valvular dehiscence, abscess, mycotic aneurysms, complete heart block, stroke, or perforations.^1,2^ Incidence of IE has increased with increasing number of valve replacements.^3,4^ It is associated with high mortality and morbidity and it ranks as the fourth most common life-threatening infection syndrome after sepsis, pneumonia, and intra-abdominal abscess.^5^

Patients with severe progressive cardiomyopathy who develop IE have limited options for advanced therapies due to risk of bacterial seeding on a left ventricular assist device (LVAD) and risk of fulminant infection in a setting of immunosuppression after heart transplantation.

We present a case of aortic valve replacement (AVR) for IE and successful bridge to heart transplantation with temporary mechanical circulatory support (MCS; Impella 5.5) that allowed for the treatment of infection as well as overall improvement in patient’s clinical condition. Our case provides additional options for clinicians when faced with limited options in managing decompensated heart failure patients with active infection, who are currently waiting for LVAD or orthotopic heart transplantation (OHT).

## Case report

This is a 62-year-old male with history of dilated bi-ventricular non-ischaemic cardiomyopathy with left ventricular ejection fraction (LVEF) of 19%, right ventricular EF of 31%, left ventricular end diastolic dimension of 7.5 cm, bicuspid aortic valve complicated by endocarditis, status post-mechanical AVR 15 years ago and bioprosthetic AVR 5 years ago, permanent pacemaker placement for complete heart block 3 years ago, and right middle cerebral artery stroke. He was recently found to have a left bundle branch block and underwent cardiac resynchronization therapy-defibrillator upgrade. A follow-up cardiac MRI revealed advanced non-ischaemic dilated cardiomyopathy without infiltrative disease. He was continued on aggressive guideline-directed medical therapy for advanced heart failure (HF).

He was admitted with progressive shortness of breath with minimal exertion, lower extremity oedema, weight gain, fatigue, and weakness. He was diagnosed with acute on chronic systolic HF exacerbation. Patient denied fever or chills. Transthoracic echocardiogram revealed EF of <20% with bi-ventricular enlargement; no valvular abnormalities were noted. Initial laboratory values were within normal limits except leucocytosis of 14.7 (4.5–11.0 × 10^9^/L), transaminitis with AST of 486 (8–33 U/L), ALT of 686 (7–56 U/L), troponin 0.214 (0.0–0.04 ng/mL), brain natriuretic peptide (BNP) 3115 (normal < 100 pg/mL), and C-reactive protein of 15 (normal < 0.5 mg/dL). Patient was borderline hypotensive and right heart catheterization demonstrated right atrial pressures of 18 mmHg, pulmonary artery pressures of 58/29/39 mmHg, and pulmonary capillary wedge pressure of 28 mmHg, with estimated Fick cardiac index of 1.7. He was found to be in cardiogenic shock with Forrester classification IV with cold and wet physiology. He was admitted to Intensive Care for inotropic support and i.v. diuresis.

**Figure 1:**
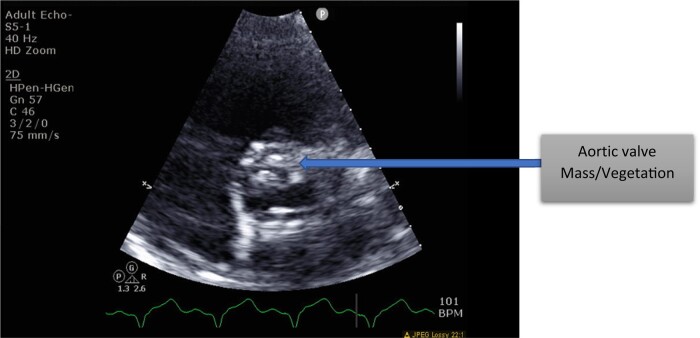
Aortic valve mass; diagnosed on endocarditis.

**Figure 2:**
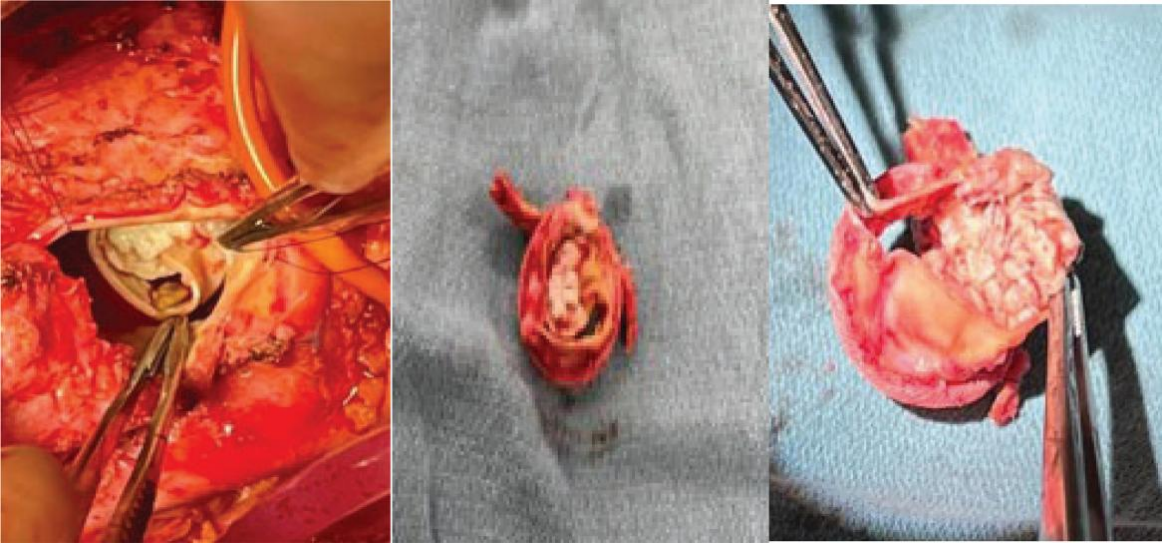
Surgical specimen of the aortic valve mass. Prosthetic aortic valve with significant vegetations noted.

**Figure 3:**
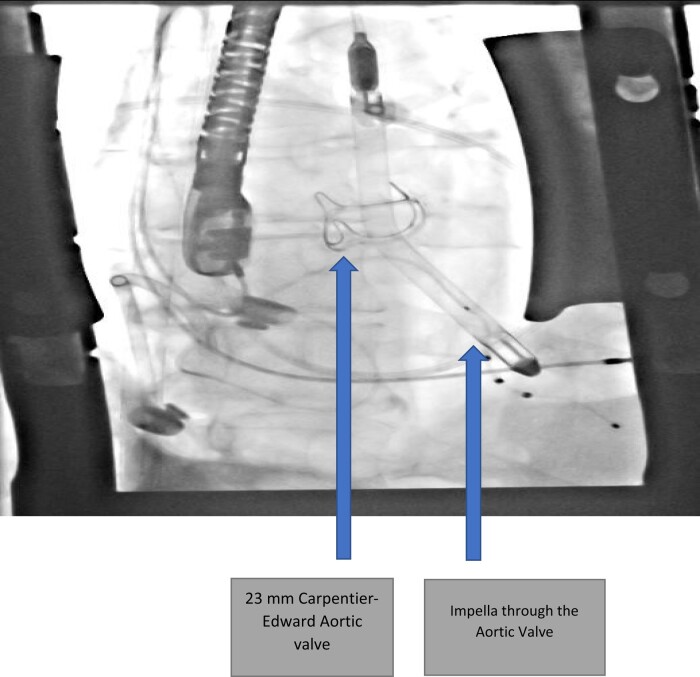
Post-operative image showing Impella through a newly placed 23 mm Carpentier-Edwards aortic valve.

**Figure 4:**
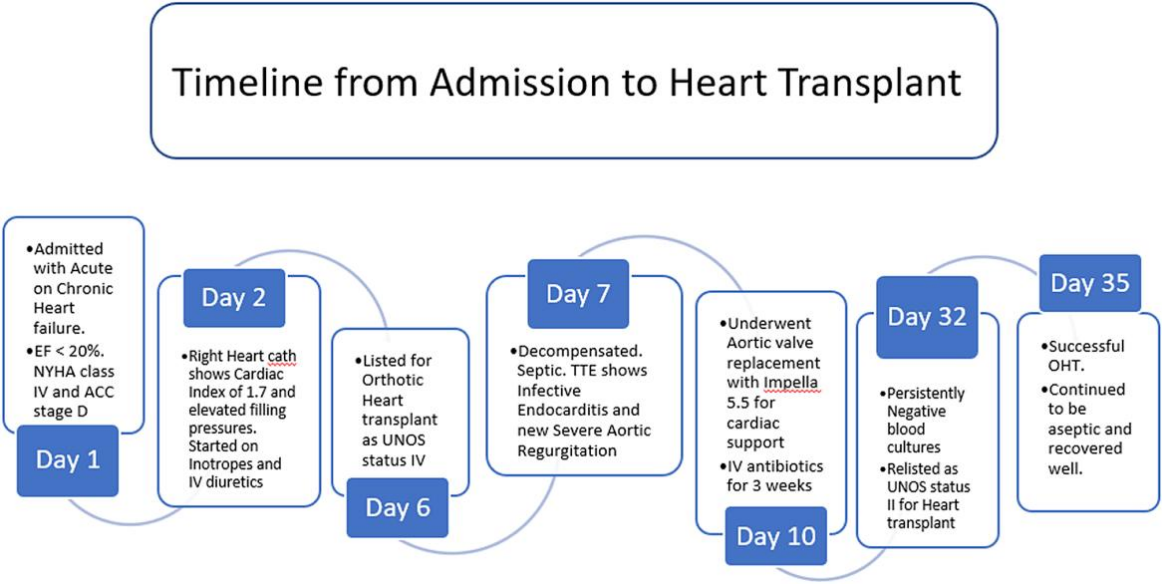
Timeline of the events from admission to heart transplant.

He was in American College of Cardiology (ACC) stage D and New York Heart Association (NYHA) class IV. We initiated evaluation for advanced heart failure therapies and the patient was listed for heart transplantation as United Network for Organ Sharing (UNOS) status 4.^6,7^ On Day 2 after listing, he decompensated further and became febrile. A repeat echocardiogram revealed a large (1.3 × 1.1 cm) echodensity on the bioprosthetic aortic valve (see *Figure 1*) and severe aortic insufficiency (AI). Blood cultures grew *Cutibacterium acnes*. He was started on vancomycin and ceftriaxone and his UNOS status was deactivated to status 7. He was in cardiogenic and septic shock requiring inotropes and vasopressors. Temporary MCS options were limited due to his severe AI and active endocarditis precluded him from intra-aortic balloon pump (IABP), veno-arterial extracorporeal membrane oxygenation (VA-ECMO), or Impella placement. Despite high Society of Thoracic Surgery (STS) risk, we proceeded with urgent AVR. At the time of surgery, the patient was found to have large vegetation on the two leaflets of the bioprosthetic aortic valve (*Figure 2*). His root was debrided for all visible foreign material and a 23 mm Carpentier-Edwards Perimount Magna Ease bioprosthetic aortic valve was placed followed by Impella 5.5 via right subclavian artery cutdown. Impella was necessary due to patient’s persistent cardiogenic shock with inability to wean off of cardiac bypass despite being on maximal inotropic and vasopressor support. Impella 5.5 via subclavian access was chosen over IABP/VA-ECMO as it enabled early mobilization of the patient. Patient was extubated and mobilized within 24 h from surgery. He was continued on i.v. antibiotics for a total of 3 weeks.^8^ Tissue cultures of aortic valve were consistent with blood cultures of *C. acnes*. His repeat blood cultures were persistently negative and he was deemed sterile prior to being reactivated on the heart transplant list as UNOS status 2.^9^ He underwent successful OHT within a week from relisting (*Figures 3* and *4*).

He was initiated on our institutional approved immunosuppression protocol. For pre-induction, he received mycophenolate and methylprednisolone followed by tacrolimus, mycophenolate, and prednisone for maintenance. He did not receive any antibody induction with basiliximab or thymoglobulin. He did not develop any recurrent infections and progressed well post-OHT. He was discharged from hospital in 2 weeks. Post-OHT work-up remained negative for infection or rejection. He has recovered completely from the multiple surgeries and continues to enjoy good health as noted at his 6- and 12-month OHT follow-up visit.

## Discussion

Infective endocarditis in prosthetic valve remains challenging despite advancements in diagnostic and treatment modalities. It has a worse prognosis if patient is in cardiogenic shock. Timing of procedures is crucial and requires an experienced team including an advanced heart failure cardiologist, cardiothoracic surgeon, interventional cardiologist, and an infectious disease specialist.

Our case reports a challenging clinical scenario of a patient with severely advanced cardiomyopathy and concomitant IE. The use of urgent valve replacement combined with temporary mechanical support to allow clearing of infection but at the same time providing adequate haemodynamic support, early extubation, mobilization, and nutrition seems to be a reasonable option in this patient population.

## Supplementary Material

ytae062_Supplementary_Data

## Data Availability

The data included in this article are available in the article and in its online supplementary material. Data will be shared on reasonable request to the corresponding author.

## Lead author biography

Advanced Heart Failure Fellow at Minneapolis Heart Institute, Minneapolis, MN.

## Supplementary material

**Consent:** Patient provided consent for his case to be published in medical literature. His questions about the basics of case reports, research, risks, and benefits of publishing were answered to his satisfaction. This was in accordance to COPE guidelines.

**Funding:** There was no funding for our case report from any source. No other author has any financial disclosures.
