# Supplementary figures and images for: Heart transplantation in a patient with infective endocarditis bridged with Impella 5.5: a case report
Source: Eur Heart J Case Rep. 2024 Mar 8;8(3):ytae062. doi: 10.1093/ehjcr/ytae062 (PMC10923287; doi:10.1093/ehjcr/ytae062)

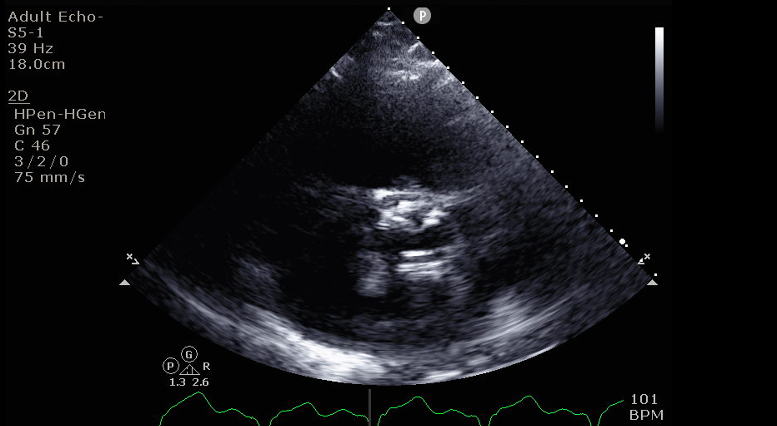


Aortic valve mass/vegetation

Supplement: ytae062_Supplementary_Data [file ytae062_supplementary_data.docx]
